# Supplementary material for: Somatic maintenance impacts the evolution of mutation rate
Source: BMC Evol Biol. 2019 Aug 23;19:172. doi: 10.1186/s12862-019-1496-y (PMC6708161; doi:10.1186/s12862-019-1496-y)
Supplement: Supplementary file 1 — File contains all the supplementary materials for the manuscript. (DOCX 500 kb) [file 12862_2019_1496_MOESM1_ESM.docx]

**Section 1. Model code.**

1. ***General model for positive selection for body size***

for iteration = 1 : 25

disp(iteration);

newrun = true;

if(newrun)

clearvars -except newrun iteration

fname = 1;

timeun = 1;

iter = 1;

% OUTPUT STORAGE MATRICES

sommortality = []; % counts of mortality for somatic reasons

extmortality = []; % counts of mortality caused by external hazard

capmortality = []; % counts of mortality imposed by ecosystem's

% carrying capacity (intra-specific competition)

biomassdyn = []; % population biomas dynamics over time

popsizedyn = []; % population size dynamics over time

births = []; % counts of new births over time

bodymassevol = []; % population's average bodymass over time

birthmassevol = []; % population's average birthmass over time

littersizeevol = []; % population's average litter size over time

mutrateevol = []; % population's average mutation rate over time

rrateevol = []; % population's average reproduction rate over time

lifespanevol = []; % population's average somatic maintenance

% coefficient over time

else

timeun = timeunit + 1;

if(fname == size(filenames, 2))

fname = 1;

else

fname = fname + 1;

end

end

filenames = ['a' 'b' 'c' 'd' 'e' 'f' 'g' 'h' 'i' 'j' 'k' 'l' 'm' 'n'...

'o' 'p' 'q' 'r' 's' 't' 'u' 'v' 'w' 'x' 'y' 'z'];

% GENERAL MODEL PARAMETERS:

totaltime = 1005000; % total # of simulation updates ("time")

popsize = 10000; % initial population size

mutrate = 0.000000001;

inhvar = 25000000; % a multiplier of mutation rate determining

% variance in trait inheritance (var=inhvar*mutrate)

% so that inhertance variance is proportional to

% mutation rate

bodymass = 5000; % initial adult bosymass

birthmass = 300; % initial body mass at birth

repbodymass = 0.9693; % multiplier determining at what body mass

% as a fraction of the individual's inherited

% adult body mass the individual begins to

% reproduce

rrate = 600; % initial time (in # simulation updates)

% between successive reproductions

littersize = 1; % initial # progeny per reproduction per individual

littervar = (0.1*littersize)/littersize; % variance of littersize

rratevar = (0.1*rrate)/rrate; % variance of reproduction rate

growthrate = 57; % coefficient of body growth rate

somdeath = 0.34; % an exponential coefficient of the somatic maintenance equation

somEnergy = 2231.81365913237; % initial energy invested in somatic

% maintenance when somdeath=0.34

% see SUPPLEMENTS

aging = mutrate*exp([1:1000000].^somdeath); % the aging function –

% probability of dying for somatic reasons over time

risk = cumsum(aging); % cumulative sum function of the aging curve

[c riskage] = min(abs(risk-1)); % riskage - age at which risk = 1;

% explained in METHODS

%INITIAL BODY GROWTH FUNCTION

growthcurve = [birthmass]; % curve for body size distribution in the initial population

% initial population is generated with ages

% ranging from 1 to riskage, they are

% aasigned their current body mass according

% to growthfunction

for i = 2 : riskage

growthcurve(i) = growthcurve(i-1) + 0.3*growthrate*(1 - (growthcurve(i-1)/bodymass));

end

[c reprodage] = min(abs(growthcurve-(bodymass*repbodymass))); % age

% of beginning to reproduce when body weight reaches

% bodymass*repbodymass (slightly smaller than adult)

repenergy = birthmass*littersize/bodymass; % a koefficient of investment

% into reproduction

% used for balancing how much energy an

% individual can invest into

% different reproductive

% parameteres

exthaz = 0.0001; % koefficient affecting the chance of dying of external

% hazards

a = 1;

b = 1; % exthaz, a and b are used in the Lotka-Volterra equation that

% regulates external hazard pressure

% INITIAL POPULATION

initpop(1, 1:popsize) = 1:popsize; %1.

initpop(2, 1:popsize) = randi([1, riskage], 1, popsize); %2.

initpop(3, 1:popsize) = ones(1, popsize).*bodymass; %3.

initpop(4, 1:popsize) = growthcurve(1, initpop(2, :)); %4.

initpop(5, 1:popsize) = birthmass; %5.

initpop(6, 1:popsize) = mutrate; %6.

initpop(7, 1:popsize) = rrate; %7.

initpop(8, 1:popsize) = littersize; %8.

initpop(9, 1:popsize) = somdeath; %9.

initpop(10, 1:popsize) = reprodage; %10.

initpop(11, 1:popsize) = somEnergy; %11.

%1. individual ID (used in mixed genotype experiments to identify genotype)

%2. current age

%3. inherited body mass

%4. current body mass

%5. inherited birth mass

%6. inherited mutation rate

%7. inherited reproduction rate

%8. inherited litter size

%9. parameter of somatic death probability function (somdeath) in the aging

% function

%10. age when beginning to reproduce

%11. energy invested in somatic maintenance (explained in METHODS)

if(newrun) % initial population is created at the beginning of simulation

population = initpop;

end

%THE CORE SIMULATION RUN

for timeunit = timeun : totaltime

disp(timeunit);

% STORAGE MATRICES KEEP TRACK OF POPULATION PARAMETERS THROUGHOUT SIMULATION

biomassdyn(timeunit) = sum(population(4, :))/sum(initpop(4, :));

popsizedyn(timeunit) = size(population, 2)/size(initpop,2);

bodymassevol(timeunit) = mean(population(3, :));

birthmassevol(timeunit) = mean(population(5, :));

littersizeevol(timeunit) = mean(population(8, :));

mutrateevol(timeunit) = mean(population(6, :));

rrateevol(timeunit) = mean(population(7, :));

lifespanevol(timeunit) = 1/mean(population(9, :));

%========================= REPRODUCTION ===========================

% potreprodpop (potentially reproducing population) collects mature

% subpopulation

potreprodpop = population(:, population(2, :)-population(10, :)>0);

% variance is introduced in time between reproductions

reprodvars = round(normrnd(rrate, rratevar));

% reprodpop (reproducing population) collects individuals that are

% past their period between reproduction and are due reproducing

% (+ some additional variance)

reprodpop = potreprodpop(:, rem(potreprodpop(2, :)-potreprodpop(10, :), reprodvars) == 0);

% copies of their parent individual are created as their progeny -

% newgen

newgen = zeros(size(reprodpop, 1), 1);

for i = 1 : size(reprodpop, 2)

if(~isempty(reprodpop))

progeny = repmat(reprodpop(1:size(reprodpop, 1), i), 1, round(normrnd(littersize, littervar)));

newgen = [newgen, progeny];

end

end

newgen = newgen(:, 2:end);

% number of new offspring is collected into a storage matrix

births(timeunit) = size(newgen, 2);

% inherited variance (proportional to parent's mutation rate)

% modifies parental parameters producing varying offspring

newgen(2, :) = 1;

newgen(3, :) = real(newgen(3, :) + (normrnd(0, newgen(6, :)*inhvar).* newgen(3, :)));

newgen(4, :) = real(newgen(5, :) + (normrnd(0, newgen(6, :)*inhvar).* newgen(5, :)));

newgen(5, :) = real(newgen(5, :) + (normrnd(0, newgen(6, :)*inhvar).* newgen(5, :)));

newgen(5, newgen(5, :) > 0.5.*newgen(3, :)) = 0.5.*newgen(3, newgen(5, :) > 0.5.*newgen(3, :));

newgen(6, :) = real(newgen(6, :) + (normrnd(0, newgen(6, :)*inhvar).* newgen(6, :)));

newgen(8, :) = real(newgen(8, :) + (normrnd(0, newgen(6, :)*inhvar).* newgen(8, :)));

newgen(7, :) = real(newgen(5, :).*newgen(8, :)./newgen(3, :)./repenergy.*rrate);

newgen(10, :) = 0;

newgen(11, :) = real(newgen(11, :) + (normrnd(0, newgen(6, :)*inhvar).* newgen(11, :)));

% the somatic maintenance (somdeath) parameter of the aging

% function is calculated based on the somatic maintenance energy

% investment with inherited variance (see METHODS)

newgen(9, :) = real((0.00000072523237903965.*(log(newgen(11, :)).^6))...

-(0.0000458064654458169.*(log(newgen(11, :)).^5))...

+(0.00123267215690707.*(log(newgen(11, :)).^4))...

-(0.0183381238349637.*(log(newgen(11, :)).^3))...

+(0.162769338153511.*(log(newgen(11, :)).^2))...

-(0.863957066277595.*(log(newgen(11, :)).^1))...

+ 2.46992883606531000000);

% new offpsring is added to the population

population = [population, newgen];

%========================= MORTALITY ===========================

%MORTALITY CAUSED BY SOMATIC/PHYSIOLOGICAL FACTORS

% individual probabilities of dying of somatic causes during this update

probsdeath = [];

% version 1 (standard) = death rates are affected by body mass

% (increased somatic risk)

% and the performance of the somtic maintenance program in

% mitigating somatic risk

probsdeath = population(6, :).*(population(4, :)/bodymass)...

.*exp(population(2, :).^population(9, :));

% version 2 = somatic cost unrelated

% (used when the "body mass" parameter is converted into

% a trait that is selected for but does not affect somatic risks)

% probsdeath = population(6, :)...

% .*exp(population(2, :).^population(9, :));

probsdeath(probsdeath > 1) = 0;

probsdeath(probsdeath < 0) = 0;

% individuals actually dying of somatic causes during this update

% based on binomial trials using probsdeath

death = [];

death = binornd(1, probsdeath(1, :));

% data on mortality of somatic causes is stored in a storage matrix

sommortality = [sommortality, population(2, death(1, :) == 1)];

%------------------------

% dead individuals are eliminated from the population

population(:, death(1, :) == 1) = 0;

population = population(:, population(1, :) > 0);

% MORTALITY CAUSED BY EXTERNAL HAZARDS (predation, disease, etc)

% (the Lotka-Voterra model of predator-prey dynamics was used as a basis)

% population size-dependent external hazard pressure (exthazard)

exthazard = exthaz...

+((a*popsizedyn(timeunit)*exthaz) - (b*exthaz));

% probabilities of dying of external hazards (development of bodymass

% or other selected trait reduces chances of dying

% of external hazards)

extprobs = [];

extprobs = exthazard.*(bodymass./population(4, :));

extprobs(1, extprobs > 1) = 1;

extprobs(1, extprobs < 0) = 0;

% individuals actually dying of causes related to external hazards

% based on binomial trials using extprobs

extdeath = [];

extdeath = binornd(1, extprobs(1, :));

% data on mortality caused by external hazards is stored in a storage matrix

extmortality = [extmortality, population(2, extdeath(1, :) == 1)];

%------------------------

% dead individuals are eliminated from the population

population(:, extdeath(1, :) == 1) = 0;

population = population(:, population(1, :) > 0);

% MORTALITY IMPOSED BY ECOSYSTEM'S CARRYING CAPACITY

% (essentially reflects mortality caused by intra-specific competition)

% Version 1 = used when maximum biomass is kept stable

% (in experiments when body mass evolves)

% (development of body mass reduces the chances of dying

% in intra-specific competition)

overkill = sum(population(4, :))/ sum(initpop(4, :));

invs = 1./population(4, :);

capprobs = invs/sum(invs);

capprobs = capprobs-(mean(capprobs));

capprobs = capprobs+(1-(1/overkill));

% Version 2 = used when population size is kept stable

% (in experiments when "body mass" is trasformed

% into another selected trait)

% (development of body mass or other selected trait

% reduces the chances of dying in intra-specific competition)

% overkill = size(population, 2) / size(initpop, 2);

% invs = 1./population(4, :);

% capprobs = invs/sum(invs);

% capprobs = capprobs-(mean(capprobs));

% capprobs = capprobs+(1-(1/overkill));

capprobs(capprobs < 0) = 0;

capprobs(capprobs > 1) = 1;

% individuals actually dying in intra-specific competitioN

% based on binomial trials using extprobs

capdeath = [];

capdeath = binornd(1, capprobs(1, :));

% data on mortality caused by intra-specific competition

% is stored in a storage matrix

capmortality = [capmortality, population(2, capdeath(1, :) == 1)];

% dead individuals are eliminated from the population

population(:, capdeath(1, :) == 1) = 0;

population = population(:, population(1, :) > 0);

% ============UPDATING AGE AND BODY MASS DUE TO GROWTH==============

population(2, :) = population(2, :) + 1;

population(4, :) = population(4, :) + 0.3*growthrate*(1 - (population(4, :)./population(3, :)));

% =============ASSIGNING MATURITY AGES FOR THE NEW OFFSPRING========

newborns = find(population(10, :) == 0);

grownnewborns = find(population(4, :)./population(3, :) >= repbodymass);

mature = intersect(newborns, grownnewborns);

population(10, mature) = population(2, mature);

% ================SAVING VARIABLES INTO FILES=======================

if(fname > size(filenames, 2))

fname = 1;

iter = iter+1;

end

if(rem(timeunit, 15000) == 0)

its(1:iter) = 'z';

save(['D:\' its filenames(fname) '.mat']);

end

if(rem(timeunit, 30000) == 0)

fname = fname + 1;

end

% =========REMOVAL OF OCCASIONAL NaNs===============================

population(:, isnan(sum(population(:, :)))) = 0;

population = population(:, population(1, :) > 0);

end

% =========ENTIRE SIMULATION RUN IS SAVED IN A FILE=====================

these(1:iteration) = '0';

save(['D:\' these 'zzh.mat']);

end

% ======TOTAL SIMULATION TIME MEASURES======================================

time = toc;

hours = floor(time / 3600);

time = time - hours * 3600;

mins = floor(time / 60);

secs = time - mins * 60;

secs = round(secs);

fprintf('Execution time (HH:MM:SS) - %d:%d:%d \n\n', hours, mins, secs);

1. ***Competitive model for competition between two genotypes*.**

for iteration = 1 : 25

disp(iteration);

newrun = true;

if(newrun)

clearvars -except newrun iteration

fname = 1;

timeun = 1;

iter = 1;

% OUTPUT STORAGE

sommortality = []; % counts of mortality for somatic reasons

extmortality = []; % counts of mortality caused by external hazard

capmortality = []; % counts of mortality imposed by ecosystem's

% carrying capacity (intra-specific competition)

biomassdyn = []; % population biomas dynamics over time

popsizedyn = []; % population size dynamics over time

births = []; % counts of new births over time

fracspec1 = []; % fraction of genotype 1

% individual parameters for genotype 1

bodymassevol1 = []; % population's average bodymass over time

birthmassevol1 = []; % population's average birthmass over time

littersizeevol1 = []; % population's average litter size over time

mutrateevol1 = []; % population's average mutation rate over time

rrateevol1 = []; % population's average reproduction rate over time

lifespanevol1 = []; % population's average somatic maintenance

% coefficient over time

% individual parameters for genotype 2

bodymassevol2 = []; % population's average bodymass over time

birthmassevol2 = []; % population's average birthmass over time

littersizeevol2 = []; % population's average litter size over time

mutrateevol2 = []; % population's average mutation rate over time

rrateevol2 = []; % population's average reproduction rate over time

lifespanevol2 = []; % population's average somatic maintenance

% coefficient over time

else

timeun = timeunit + 1;

if(fname == size(filenames, 2))

fname = 1;

else

fname = fname + 1;

end

end

filenames = ['a' 'b' 'c' 'd' 'e' 'f' 'g' 'h' 'i' 'j' 'k' 'l' 'm' 'n'...

'o' 'p' 'q' 'r' 's' 't' 'u' 'v' 'w' 'x' 'y' 'z'];

% GENERAL MODEL PARAMETERS:

totaltime = 1005000; % total # of simulation updates ("time")

popsize = 10000; % initial population size

mutrate = 0.000000001;

inhvar = 25000000; % a multiplier of mutation rate determining

% variance in trait inheritance (var=inhvar*mutrate)

% so that inhertance variance is proportional to

% mutation rate

bodymass = 5000; % initial adult bosymass

birthmass = 300; % initial body mass at birth

repbodymass = 0.9693; % multiplier determining at what body mass

% as a fraction of the individual's inherited

% adult body mass the individual begins to

% reproduce

rrate = 600; % initial time (in # simulation updates)

% between successive reproductions

littersize = 1; % initial # progeny per reproduction per individual

littervar = (0.1*littersize)/littersize; % variance of littersize

rratevar = (0.1*rrate)/rrate; % variance of reproduction rate

growthrate = 57; % coefficient of body growth rate

somdeath = 0.34; % an exponential coefficient of the somatic maintenance equation

somEnergy = 2231.81365913237; % initial energy invested in somatic

% maintenance when somdeath=0.34

% see SUPPLEMENTS

aging = mutrate*exp([1:1000000].^somdeath); % the aging function –

% probability of dying for somatic reasons over time

risk = cumsum(aging); % cumulative sum function of the aging curve

[c riskage] = min(abs(risk-1)); % riskage - age at which risk = 1;

% explained in METHODS

%INITIAL BODY GROWTH FUNCTION

growthcurve = [birthmass]; % curve for body size distribution in the initial population

% initial population is generated with ages

% ranging from 1 to riskage, they are

% aasigned their current body mass according

% to growthfunction

for i = 2 : riskage

growthcurve(i) = growthcurve(i-1) + 0.3*growthrate*(1 - (growthcurve(i-1)/bodymass));

end

[c reprodage] = min(abs(growthcurve-(bodymass*repbodymass))); % age

% of beginning to reproduce when body weight reaches

% bodymass*repbodymass (slightly smaller than adult)

repenergy = birthmass*littersize/bodymass; % a koefficient of investment

% into reproduction

% used for balancing how much energy an

% individual can invest into

% different reproductive

% parameteres

exthaz = 0.0001; % koefficient affecting the chance of dying of external

% hazards

a = 1;

b = 1; % exthaz, a and b are used in the Lotka-Volterra equation that

% regulates external hazard pressure

%initial population

initpop(1, 1:ceil(popsize/2)) = 1; %1 genotype 1

initpop(1, ceil(popsize/2)+1:popsize) = 2; %1 genotype 2

initpop(2, 1:popsize) = randi([1, maxage], 1, popsize); %2

initpop(3, 1:popsize) = ones(1, popsize).*bodymass; %3

initpop(4, 1:popsize) = growthcurve(1, initpop(2, :)); %4

initpop(5, 1:popsize) = birthmass; %5

initpop(6, 1:ceil(popsize/2)) = mutrate/10; %6 genotype 1

initpop(6, ceil(popsize/2)+1:popsize) = mutrate; %6 genotype 2

initpop(7, 1:popsize) = rrate; %7

initpop(8, 1:popsize) = littersize; %8

initpop(9, 1:popsize) = somdeath; %9

initpop(10, 1:popsize) = reprodage; %10

initpop(11, 1:popsize) = somEnergy; %11

%1. individual ID (used in mixed genotype experiments to identify genotype)

%2. current age

%3. inherited body mass

%4. current body mass

%5. inherited birth mass

%6. inherited mutation rate

%7. inherited reproduction rate

%8. inherited litter size

%9. parameter of somatic death probability function (somdeath) in the aging

% function

%10. age when beginning to reproduce

%11. energy invested in somatic maintenance (explained in METHODS)

if(newrun) % initial population is created at the beginning of simulation

population = initpop;

end

%THE CORE SIMULATION RUN

for timeunit = timeun : totaltime

disp(timeunit);

% STORAGE MATRICES KEEP TRACK OF POPULATION PARAMETERS THROUGHOUT SIMULATION

biomassdyn(timeunit) = sum(population(4, :))/sum(initpop(4, :));

popsizedyn(timeunit) = size(population, 2)/size(initpop,2);

bodymassevol1(timeunit) = mean(population(3, population(1,:)==1));

birthmassevol1(timeunit) = mean(population(5, population(1,:)==1));

littersizeevol1(timeunit) = mean(population(8, population(1,:)==1));

mutrateevol1(timeunit) = mean(population(6, population(1,:)==1));

rrateevol1(timeunit) = mean(population(7, population(1,:)==1));

lifespanevol1(timeunit) = 1/mean(population(9, population(1,:)==1));

bodymassevol2(timeunit) = mean(population(3, population(1,:)==2));

birthmassevol2(timeunit) = mean(population(5, population(1,:)==2));

littersizeevol2(timeunit) = mean(population(8, population(1,:)==2));

mutrateevol2(timeunit) = mean(population(6, population(1,:)==2));

rrateevol2(timeunit) = mean(population(7, population(1,:)==2));

lifespanevol2(timeunit) = 1/mean(population(9, population(1,:)==2));

fracspec1(timeunit) = numel(population(1, population(1,:) == 1))/size(population, 2)*100;

%========================= REPRODUCTION ===========================

% potreprodpop (potentially reproducing population) collects mature

% subpopulation

potreprodpop = population(:, population(2, :)-population(10, :)>0);

% variance is introduced in time between reproductions

reprodvars = round(normrnd(rrate, rratevar));

% reprodpop (reproducing population) collects individuals that are

% past their period between reproduction and are due reproducing

% (+ some additional variance)

reprodpop = potreprodpop(:, rem(potreprodpop(2, :)-potreprodpop(10, :), reprodvars) == 0);

% copies of their parent individual are created as their progeny -

% newgen

newgen = zeros(size(reprodpop, 1), 1);

for i = 1 : size(reprodpop, 2)

if(~isempty(reprodpop))

progeny = repmat(reprodpop(1:size(reprodpop, 1), i), 1, round(normrnd(littersize, littervar)));

newgen = [newgen, progeny];

end

end

newgen = newgen(:, 2:end);

% number of new offspring is collected into a storage matrix

births(timeunit) = size(newgen, 2);

% inherited variance (proportional to parent's mutation rate)

% modifies parental parameters producing varying offspring

newgen(2, :) = 1;

newgen(3, :) = real(newgen(3, :) + (normrnd(0, newgen(6, :)*inhvar).* newgen(3, :)));

newgen(4, :) = real(newgen(5, :) + (normrnd(0, newgen(6, :)*inhvar).* newgen(5, :)));

newgen(5, :) = real(newgen(5, :) + (normrnd(0, newgen(6, :)*inhvar).* newgen(5, :)));

newgen(5, newgen(5, :) > 0.5.*newgen(3, :)) = 0.5.*newgen(3, newgen(5, :) > 0.5.*newgen(3, :));

% mutation rates are fixed and differ between two genotypes

% newgen(6, newgen(1, :) == 1) = real(newgen(6, newgen(1, :) == 1) + (normrnd(0, newgen(6, newgen(1, :) == 1)*inhvar).* newgen(6, newgen(1, :) == 1)));

newgen(8, :) = real(newgen(8, :) + (normrnd(0, newgen(6, :)*inhvar).* newgen(8, :)));

newgen(7, :) = real(newgen(5, :).*newgen(8, :)./newgen(3, :)./repenergy.*rrate);

newgen(10, :) = 0;

newgen(11, :) = real(newgen(11, :) + (normrnd(0, newgen(6, :)*inhvar).* newgen(11, :)));

% the somatic maintenance (somdeath) parameter of the aging

% function is calculated based on the somatic maintenance energy

% investment with inherited variance (see METHODS)

newgen(9, :) = real((0.00000072523237903965.*(log(newgen(11, :)).^6))...

-(0.0000458064654458169.*(log(newgen(11, :)).^5))...

+(0.00123267215690707.*(log(newgen(11, :)).^4))...

-(0.0183381238349637.*(log(newgen(11, :)).^3))...

+(0.162769338153511.*(log(newgen(11, :)).^2))...

-(0.863957066277595.*(log(newgen(11, :)).^1))...

+ 2.46992883606531000000);

% new offpsring is added to the population

population = [population, newgen];

%========================= MORTALITY ===========================

%MORTALITY CAUSED BY SOMATIC/PHYSIOLOGICAL FACTORS

% individual probabilities of dying of somatic causes during this update

probsdeath = [];

% version 1 (standard) = death rates are affected by body mass

% (increased somatic risk)

% and the performance of the somtic maintenance program in

% mitigating somatic risk

probsdeath = population(6, :).*(population(4, :)/bodymass)...

.*exp(population(2, :).^population(9, :));

% version 2 = somatic cost unrelated

% (used when the "body mass" parameter is converted into

% a trait that is selected for but does not affect somatic risks)

% probsdeath = population(6, :)...

% .*exp(population(2, :).^population(9, :));

probsdeath(probsdeath > 1) = 0;

probsdeath(probsdeath < 0) = 0;

% individuals actually dying of somatic causes during this update

% based on binomial trials using probsdeath

death = [];

death = binornd(1, probsdeath(1, :));

% data on mortality of somatic causes is stored in a storage matrix

sommortality = [sommortality, population(2, death(1, :) == 1)];

%------------------------

% dead individuals are eliminated from the population

population(:, death(1, :) == 1) = 0;

population = population(:, population(1, :) > 0);

% MORTALITY CAUSED BY EXTERNAL HAZARDS (predation, disease, etc)

% (the Lotka-Voterra model of predator-prey dynamics was used as a basis)

% population size-dependent external hazard pressure (exthazard)

exthazard = exthaz...

+((a*popsizedyn(timeunit)*exthaz) - (b*exthaz));

% probabilities of dying of external hazards (development of bodymass

% or other selected trait reduces chances of dying

% of external hazards)

extprobs = [];

extprobs = exthazard.*(bodymass./population(4, :));

extprobs(1, extprobs > 1) = 1;

extprobs(1, extprobs < 0) = 0;

% individuals actually dying of causes related to external hazards

% based on binomial trials using extprobs

extdeath = [];

extdeath = binornd(1, extprobs(1, :));

% data on mortality caused by external hazards is stored in a storage matrix

extmortality = [extmortality, population(2, extdeath(1, :) == 1)];

%------------------------

% dead individuals are eliminated from the population

population(:, extdeath(1, :) == 1) = 0;

population = population(:, population(1, :) > 0);

% MORTALITY IMPOSED BY ECOSYSTEM'S CARRYING CAPACITY

% (essentially reflects mortality caused by intra-specific competition)

% Version 1 = used when maximum biomass is kept stable

% (in experiments when body mass evolves)

% (development of body mass reduces the chances of dying

% in intra-specific competition)

overkill = sum(population(4, :))/ sum(initpop(4, :));

invs = 1./population(4, :);

capprobs = invs/sum(invs);

capprobs = capprobs-(mean(capprobs));

capprobs = capprobs+(1-(1/overkill));

% Version 2 = used when population size is kept stable

% (in experiments when "body mass" is trasformed

% into another selected trait)

% (development of body mass or other selected trait

% reduces the chances of dying in intra-specific competition)

% overkill = size(population, 2) / size(initpop, 2);

% invs = 1./population(4, :);

% capprobs = invs/sum(invs);

% capprobs = capprobs-(mean(capprobs));

% capprobs = capprobs+(1-(1/overkill));

capprobs(capprobs < 0) = 0;

capprobs(capprobs > 1) = 1;

% individuals actually dying in intra-specific competitioN

% based on binomial trials using extprobs

capdeath = [];

capdeath = binornd(1, capprobs(1, :));

% data on mortality caused by intra-specific competition

% is stored in a storage matrix

capmortality = [capmortality, population(2, capdeath(1, :) == 1)];

% dead individuals are eliminated from the population

population(:, capdeath(1, :) == 1) = 0;

population = population(:, population(1, :) > 0);

% ============UPDATING AGE AND BODY MASS DUE TO GROWTH==============

population(2, :) = population(2, :) + 1;

population(4, :) = population(4, :) + 0.3*growthrate*(1 - (population(4, :)./population(3, :)));

% =============ASSIGNING MATURITY AGES FOR THE NEW OFFSPRING========

newborns = find(population(10, :) == 0);

grownnewborns = find(population(4, :)./population(3, :) >= repbodymass);

mature = intersect(newborns, grownnewborns);

population(10, mature) = population(2, mature);

% ================SAVING VARIABLES INTO FILES=======================

if(fname > size(filenames, 2))

fname = 1;

iter = iter+1;

end

if(rem(timeunit, 15000) == 0)

its(1:iter) = 'z';

save(['D:\' its filenames(fname) '.mat']);

end

if(rem(timeunit, 30000) == 0)

fname = fname + 1;

end

% =========REMOVAL OF OCCASIONAL NaNs===============================

population(:, isnan(sum(population(:, :)))) = 0;

population = population(:, population(1, :) > 0);

end

% =========ENTIRE SIMULATION RUN IS SAVED IN A FILE=====================

these(1:iteration) = '0';

save(['D:\' these 'zzh.mat']);

end

% ======TOTAL SIMULATION TIME MEASURES======================================

time = toc;

hours = floor(time / 3600);

time = time - hours * 3600;

mins = floor(time / 60);

secs = time - mins * 60;

secs = round(secs);

fprintf('Execution time (HH:MM:SS) - %d:%d:%d \n\n', hours, mins, secs);

**Section 2. Evolution of reproductive traits under fixed adult body mass.** As shown in **Fig. S1**, the early simulation period is linked with rapid evolution of reproduction rate and body mass at birth, which is likely to have caused positive selection for gMR shown in **Fig. 3B**. Litter size, however, in our simulations did not show any consistent evolution under this condition.


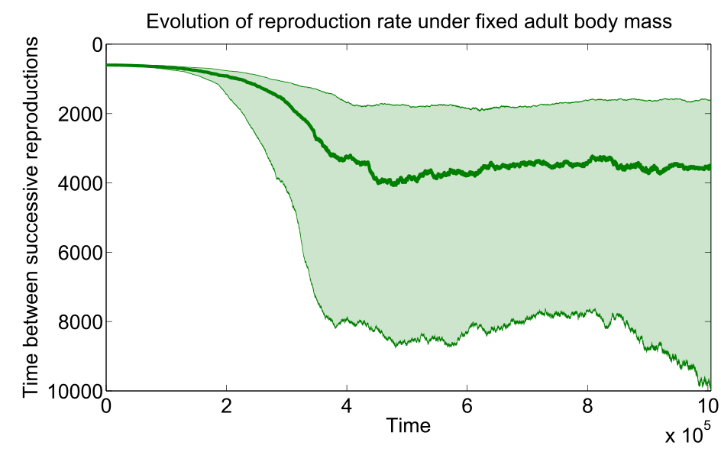

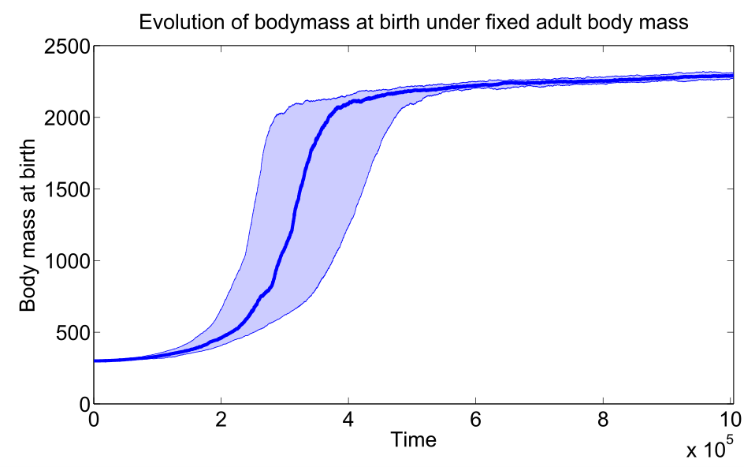


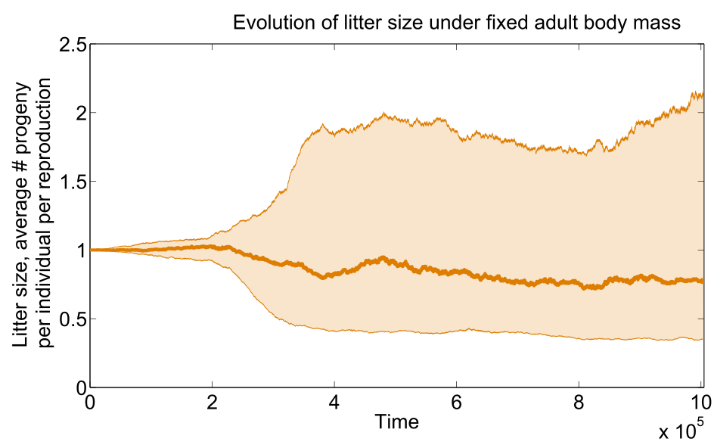


**Fig. S1. Evolution of reproductive parameters in simulations with fixed adult body mass.**

**Section 3. All-cause age-dependent mortality in the model.** The model recapitulates a typical age-dependent mortality chart for wild animals (**Fig. S2**). Early life is accompanied with the very high mortality rates which drop until maturity. **Fig. S3** demonstrates natural log data.


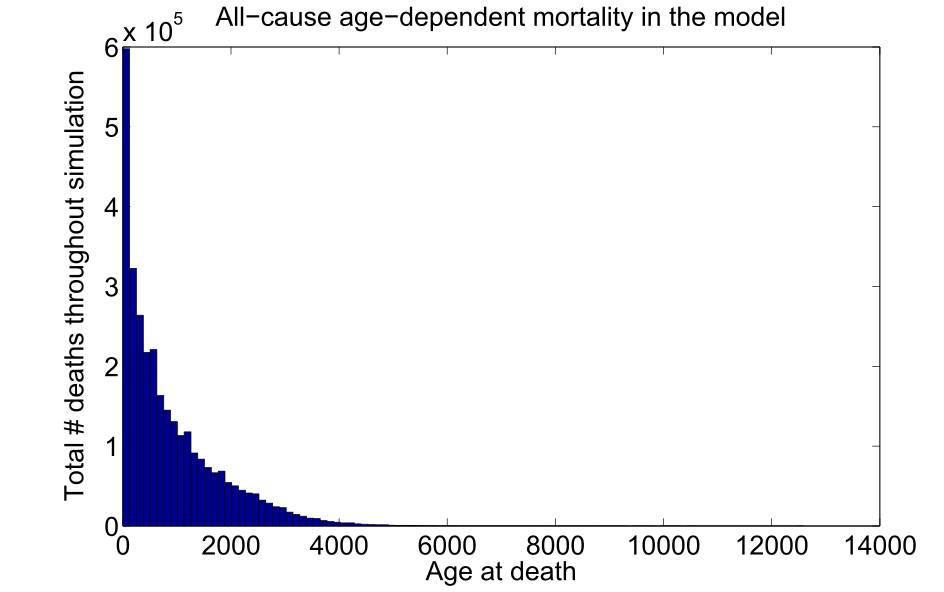


**Fig. S2. Total mortality by age in absolute numbers.**


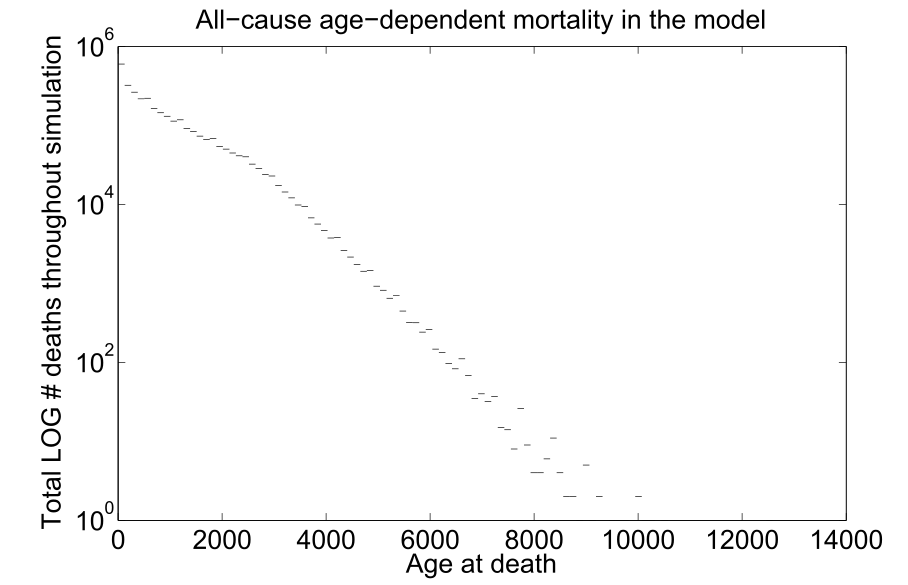


**Fig. S3. Total mortality by age in log-absolute numbers.**

**Section 4. The aging curve calculations.** In order to model inherited variation of SMP strength, we needed a method of varying the SMP curve so that an individual’s efficiency of SMP can be inherited with normally distributed variation (e.g. +1%, -5% etc). Since the *Som* parameter in **Eq. 1** is in a complex non-linear relationship with the resulting aging curve, this parameter is not suitable for such manipulation. We therefore reasoned that the best representation of the efficiency of SMP is using the area under the physiological mortality curve as a measure of the general efficiency of SMP over lifetime. **Eq. 1** generates the probability *D_A_* of dying of physiological causes at age A. Its cumulative probability function generates probability *D(A)* of dying by age A. *D(A)* thus is directly related with longevity (like the human mortality curve). In the example shown in **Fig. S4**, the green curve represents extended longevity compared to the blue curve, since the cumulative probability of dying by time T (green shaded area) grows more slowly (slower aging). As a result, the area under the green curve is larger, corresponding to a stronger SMP program. In order to model inherited variation in SMP, we used this area as a representation of the SMP strength. The area was stochastically varied from generation to generation as explained in **Methods**, and its new value in progeny was used to calculate the *Som* parameter for **Eq. 1** (determines the probability of dying at age A). The calculation was based on the observation that the area shown in **Fig. S4** demonstrates a strong non-linear log-log relationship with the *Som* parameter (polynomial regression of the 6^th^ order; R^2^>0.99999) as shown in **Fig. S5**.

**
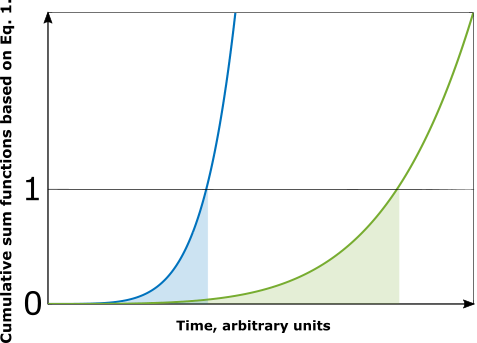
**

**Fig. S4. Area under cumulative sum function of Eq. 1 as a measure of the relative efficiency of SMP.**

**
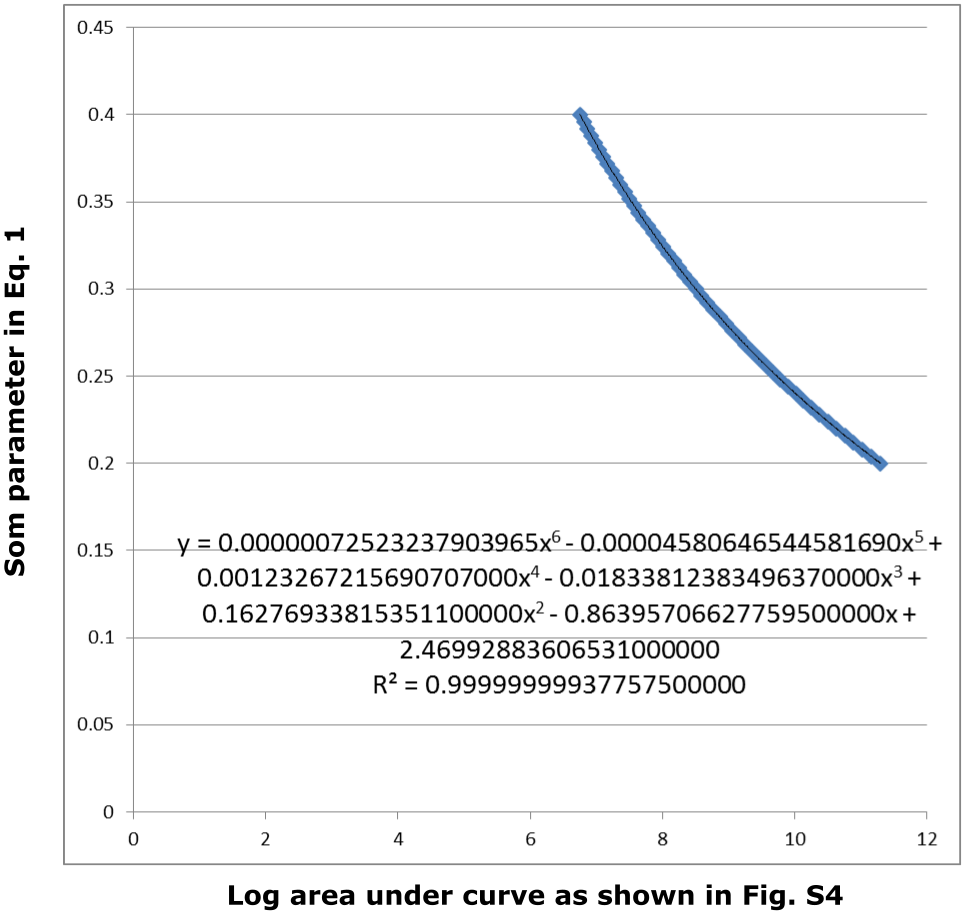
**

**Fig. S5. Relationship between log-area in Fig. S4 (log-efficiency of SMP) and the *Som* parameter in Eq. 1 (probability of dying at age A).**

**Section 5. Removal of outliers.** Occasionally the model demonstrated unnatural “spikes” in the evolution of some traits under some conditions. We had to apply the following code to remove them:

input = aaccbmbllsev_lit(22,:); % a certain problematic model run

threshold = 0.15; % arbitrary value

for row = 1 : size(input, 1)

for col = 2 : size(input, 2)

if input(row, col-1)/input(row, col) > 1+threshold ||...

input(row, col-1)/input(row, col) < 1-threshold

input(row, col) = input(row, col-1);

end

end

end

The illustration below demonstrates an example (from a standard condition run) of the result:


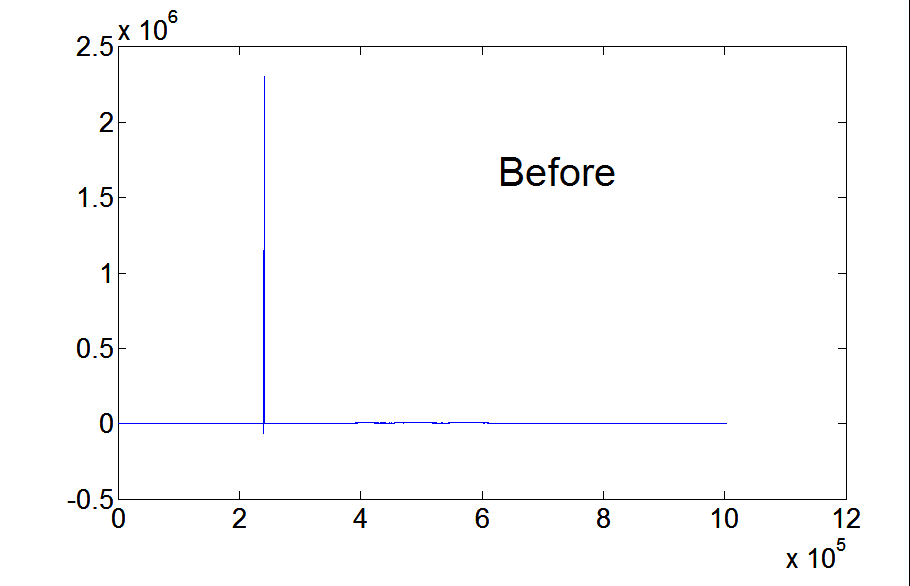

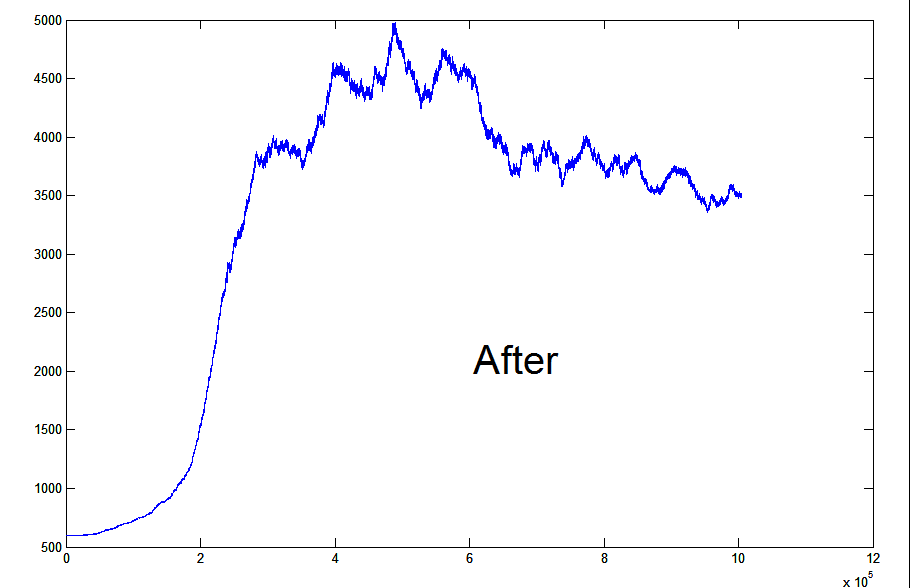


The parameter “threshhold” required manual alteration until the spike was cleaned by the code above. Such spikes were visibly outstanding from the normal trend, so that the trend in the evolving trait continued after the spike with values similar to those immediately preceding the spike, indicating that the spikes were some artifacts that neither related to nor influenced the modeled trait evolution. We were not able to determine the source of such spikes

**Section 6. Matlab code for generating the distribution of phenotypic trait expression with different number of genes encoding a trait in a population of 100,000 individuals.**

% population of phenotypes

vals = [];

genes = 7;

alleles = 8;

% for each individual phenotype is created as the average

%contribution of N genes each represented by M alleles

for i = 1 : 100000

    phen = [];

    for g = 1 : genes

        phen(g) = randi(alleles);

    end

    vals(i) = mean(phen);

end

hist(vals, 100)
